# Examining public knowledge, attitudes and perceptions towards palliative care: a mixed method sequential study

**DOI:** 10.1186/s12904-021-00730-5

**Published:** 2021-03-17

**Authors:** Sonja McIlfatrick, Paul Slater, Esther Beck, Olufikayo Bamidele, Sharon McCloskey, Karen Carr, Deborah Muldrew, Lisa Hanna-Trainor, Felicity Hasson

**Affiliations:** 1 Institute of Nursing and Health Research, School of Nursing, Ulster University, Shore Road Newtownabbey, Antrim, BT37 0QB Northern Ireland; 2 Institute of Clinical and Applied Health Research, Hull York Medical School, Allam Medical Building, University of Hull, Hull, HU6 7RX UK; 3 Southern Health and Social Care Trust, The Rowans, Craigavon Area Hospital, Lurgan Road, Portadown, BT36 5QQ Northern Ireland

**Keywords:** Palliative care, Knowledge, Attitudes, Public, Public health, Mixed methods, Health promotion

## Abstract

**Background:**

Palliative care is recognised as a public health issue with the need for earlier integration in the wider healthcare system. However, research indicates that it continues to be accessed late in the course of an illness, public understanding of palliative care is limited, and common misconceptions prevail. Strategies to address this are needed in order to reduce barriers to palliative care delivery and improve access.

**Methods:**

An explanatory sequential mixed methods study, comprising a cross-sectional survey and interviews was undertaken. Sociodemographic characteristics, public awareness, knowledge and perceptions of palliative care were examined and strategies to raise awareness and overcome barriers within a public health framework were identified. Survey data were analysed using SPSS v25 with factor analysis and non-parametric statistics and qualitative data were analysed using thematic analysis.

**Results:**

A total of 1201 participants completed the survey (58.3% female, mean age 61 years) and 25 took part in interviews. A fifth of participants (20.1%) had previously heard about palliative care and had an accurate understanding of the term. Being female, higher educated, married, and older, increased respondents’ levels of awareness. The three most commonly held misconceptions included: Palliative care is exclusively for people who are in the last 6 months of life (55.4% answered incorrectly); A goal of palliative care is to address any psychological issues brought up by serious illness (42.2% answered incorrectly); and a goal of palliative care is to improve a person’s ability to participate in daily activities (39.6% answered incorrectly). Talking about palliative and end of life care was advocated but societal taboos restricted this occurring with exposure limited to personal experience.

**Conclusions:**

Current knowledge gaps and misconceptions derived from limited ad hoc personal experiences and fear of engaging in taboo conversations may deter people from accessing integrated palliative care services early in a disease trajectory. The results indicate the need for public education programmes that move beyond merely raising awareness but provide key messages within a public health approach, which may change attitudes to palliative care thus ultimately improving end of life outcomes.

**Supplementary Information:**

The online version contains supplementary material available at 10.1186/s12904-021-00730-5.

## Background

Globally, people are living longer, and many are living with complex, chronic conditions (World Health Organisation [1, 2]. It is estimated that by 2060 there will be an 87% increase in the number of people dying with serious health-related suffering, and immediate global action is required to integrate palliative care into health systems [3]. Over the last two decades, the WHO advocated that palliative care should be considered as a public health issue, with calls for earlier integration of palliative care within the wider healthcare system to improve access and availability [1, 4, 5]. Integration of palliative care into other parts of the health system, and more broadly into society itself, is supported by a recent article in the Lancet [6]. This earlier or ‘integrated’ model of palliative care enables palliative care professionals to build relationships and become increasingly responsive to the need of patients and their families [7]. Despite this recommendation, evidence repeatedly demonstrates that palliative care is accessed late in the illness course [8]. What the public know and understand about palliative care may impact on future access to quality care in the event of a serious illness [9]. However, international research suggests that palliative care is poorly understood among the general public with varying levels of awareness and understanding of palliative care globally (Table 1), for example, there are misconceptions that palliative care is provided at the very end of life [11, 15, 26], and variable knowledge of palliative care by the public reported in America [20, 21]; Saudi Arabia [24]; Nigeria [27]; Italy [12]; Sweden [22]; and the UK [10, 15, 16, 28]. This literature confirms inconsistencies internationally, despite global efforts to increase awareness. A recent scoping review of the literature noted that thirteen international studies had been undertaken between 2003 and 2019 and concluded that the public had poor knowledge and misconceptions about palliative care [29]. Thus, there is a growing need to raise awareness and understanding of palliative care as a key public health priority [6, 30].

**Table 1:** Summary of literature on the public’s knowledge or awareness of PC

Author (Country of Origin), Year	Aim	Method, Sample Size	Findings
Wallace (Scotland), 2003 [10]	To investigate pubic knowledge and understanding of PC	Postal Survey, *n* = 668	32% had no knowledge and 49% had some knowledge of PC. Believed PC to be for patients with a terminal diagnosis of cancer
Claxton-Oldfield (Canada), 2004 [11]	To evaluate people’s understanding of PC Atlantic Canada	In person survey, *n* = 89	75.3% had never heard of PC. Believed PC to be for patients with a terminal diagnosis of cancer
Benini et al. (Italy), 2011 [12]	To examine the awareness of PC among Italians and their perception of the needs of patients with incurable illness	In person survey, *n* = 1897	40.6% had never heard of PC. Believed it to be non-curative, for terminal patients, and it improves QoL
Hirai et al. (Japan), 2011 [13]	To explore public awareness, knowledge, and readiness for PC services	Survey, *n* = 3984	63.1% had no knowledge of PC, and only 0.5% were using PC services. 18.6% knew about PC but did not know their availability
MacLoed et al. (New Zealand), 2012 [14]	To investigate New Zealanders views of and local hospice	Online survey, *n* = 1011	Reasonable understanding of PC reported, seen to provide comfort to people with terminal illness
McIlfatrick et al. (UK), 2013 [15]	To establish awareness and attitudes of the general public in Northern Ireland towards PC	Online and postal survey, *n* = 600	75% had little/no knowledge of PC, 83% had never heard of PC. Associated with older people and cancer with the aim of achieving a peaceful death
McIlfatrick et al. (UK), 2014 [16]	To explore public perceptions of PC and identify strategies to raise awareness	Semi-structured telephone survey, *n* = 50	Most had a general knowledge of PC, generally associated with dying and cancer.
Boucher et al. (USA), 2018 [17]	To understand participant knowledge PC and acceptability a new community based PC model	Focus Groups *n* = 18	Participants had varying knowledge of PC
Roulston et al. (Canada), 2018 [18]	To gauge Canadian views on PC	Online survey, *n* = 1540	43% were “somewhat aware” of PC
Yim et al. (Korea), 2018 [19]	To navigate public awareness of PC	Online survey, *n* = 1500	60.5% had no knowledge of PC
Koslov et al. (USA), 2018 [20]	To measure PC knowledge in laypersons and how different socioeconomic groups perform on PaCKS	Online survey, *n* = 301	Participants had poor knowledge of PC, with an average score < 50% on PaCKS.
Shalev et al. (USA), 2018) [21]	To examine palliative and hospice care awareness, misperceptions, and receptivity among community-dwelling adults	Telephone survey, *n* = 800	73% were unable to define PC. > 50% had at least one misperception, most commonly was to associate PC with EOL care
Westerlund et (Sweden), 2018 [22]	To investigate awareness of PC in general Swedish population	Online survey, *n* = 2020	41% had no awareness of PC and 43% had some awareness.
AbdulRaheem et al., (Nigeria), 2019 [23]	To establish current levels of awareness attitudes towards PC among the general public in Nigeria	Survey, *n* = 564	Knowledge came from personal experience working in healthcare or using PC. Gender (female) and previous experience positively influenced awareness
Alkhudairi (Saudi Arabia), 2019 [24]	To evaluate awareness, knowledge, and beliefs of the Saudi adult population regarding	Online survey, *n* = 1987	16.2% knew what PC was, 22.8% had heard of PC, and 34.4% believed PC can reduce physical suffering
Huo et al. (USA), 2019 [25]	To examine knowledge penetration of PC in adults	Survey, *n* = 3194	71% had no knowledge of PC
Taber (USA), 2019 [26]	To explore knowledge and beliefs about PC among the general public	Survey, *n* = 1162	Respondents who perceived themselves to know a lot about PC were often no less likely to report inaccurate knowledge or negative beliefs (versus accurate and positive, respectively).

A range of terms have been used to describe a public health approach to palliative care, including compassionate cities, compassionate communities, health promoting palliative care, and community development, engagement or participation [31]. Furthermore, there remains debate within the academic literature around various ‘models’ and conceptual underpinnings for this approach [30]. Three overlapping approaches to public health and palliative care have been noted in the literature: WHO approach (practice driven); Health promotion approach (community assets) and Population based approach (epidemiology) [32]. Further investigation of the public’s views of palliative care is vital to help inform and target a future public education programme that provide key messages within a public health approach, which may change attitudes to palliative care thus ultimately improving access and end of life outcomes.

### Aim

To examine public awareness, knowledge, and perceptions of palliative care and identify strategies to raise awareness within a public health framework.

## Methods

### Design

**Fig. 1:**
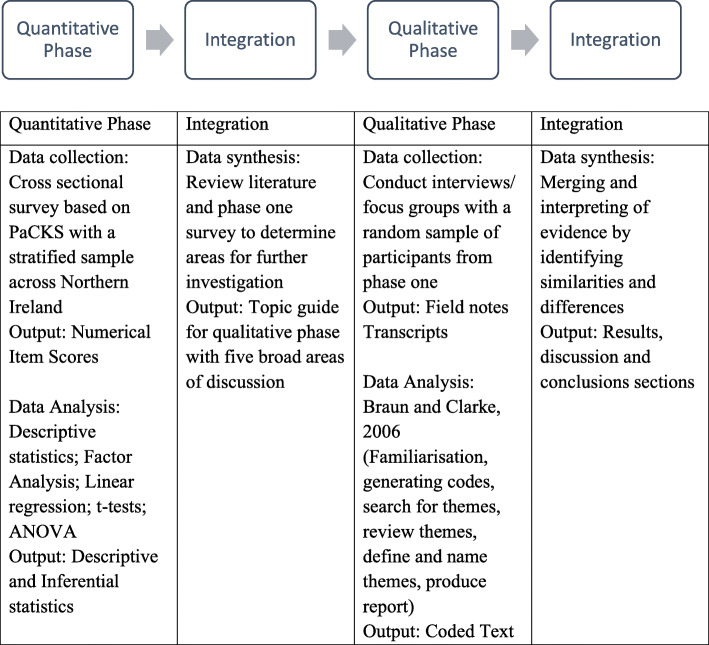
Research design

A two phase, explanatory, sequential mixed methods design based on the taxonomy of Creswell and Plano Clark [33] was utilised, including a cross-sectional survey and qualitative interviews/focus groups with members of the public (Fig. 1). The first phase assessed the knowledge, attitudes and behaviours of the public in Northern Ireland regarding palliative care, and the second phase explored these concepts in depth to give meaning to the numerical data and identify future strategies for public promotion of palliative care.

### Participants

Participants for the survey included a random representative sample of adults from the Northern Ireland population aged 18 years and over; selected from a database of addresses, where interviewers selected one adult at random for face to face completion of the survey at each address using the ‘next birthday’ method. This is an efficient method for selecting representative respondents within a household unit [34]. Participation was voluntary.

Following completion of the survey, participants were asked if they would like to participate in the second, qualitative phase. Those who agreed to contribute had their contact information collated (separately to the survey responses). Willing participants were contacted via telephone. Inclusion criteria included participant aged 18–80 years; able to speak and read English; had previously completed the survey and were willing to participate, able to provide informed consent; and not have suffered bereavement within the last 6 months. Participants who met the eligibility criteria were sent study information and upon receipt of the consent form, were invited to take part in a one-off focus group or telephone/face to face interview.

### Data collection

Phase one quantitative data was collected in 2018, as part of the cross-sectional attitudinal survey undertaken annually in Northern Ireland, known as the Northern Ireland Life and Times survey [35] (https://www.ark.ac.uk/nilt/). The NILT uses a random sample with 1200 adults completing the questionnaire each year, ensuring the socio-economic and demographic characteristics of the sample are representative of the total population [36]. This survey is run on a modular format and four modules are included each year. One module assessed the knowledge, attitudes, and behaviour of the Northern Ireland public’s towards palliative care. This is the first time palliative care has been included in this survey since the NILT began in 1998. Questions investigating palliative care were based on Palliative Care Knowledge Scale (PaCKS) [37, 38], a 13-item instrument, and true/false format that assesses goals, timing and what constitutes the Palliative care team and systems related issues [37, 38]. Seven yes/no items on participants’ prior knowledge of palliative care were also included within the NILT survey. Participant’s sociodemographic characteristics such as age, gender, religion, education level, marital status and income etc. are collected as part of the NILT annual survey (see Supplementary file 1). Face-to-face questionnaires were carried out using computer assisted personal interviewing, and there was a further self-completion questionnaire which respondents are asked to fill in on a tablet, or on paper.

In phase two, data was collected from October 2018 to July 2019 by DM, KC, EB and SMcC (postdoctoral researchers and/or specialist practitioners). The researcher was not known to participants prior to the data collection. The qualitative interview schedule comprised of five broad topic areas, which included questions on the participant’s knowledge of palliative care; whether they would seek knowledge and information on palliative care; their perceived accessibility of palliative care services and finally, future strategies including supporting and inhibiting factors for promoting public awareness of palliative care. The interview schedule was informed by the literature and quantitative phase of the study (see Supplementary file 2). While focus groups were offered, the majority of participants wanted to undertake interviews. Data were collected in a neutral public place, the participant’s home or via telephone, and were audio recorded and field notes taken. The data collection tools were piloted with academics and NILT data collectors prior to implementation.

### Data analysis

In phase one, the number of correct responses were tallied, and scores ranged from 0 (lowest knowledge) to 13 (highest knowledge). “I don’t know”/ “Not sure” responses were coded as incorrect when calculating total PaCKS scores. All survey data were analysed in SPSS v 25.0. Descriptive statistics were used to summarise the participants’ demographic factors and other variables, including participants’ prior knowledge of palliative care. A factor analysis was undertaken on PaCKS and the factor structure was found to be acceptable. Linear regression was used to identify demographic variables that impacted on the PaCKS score and explored further using inferential statistics. Further analyses were undertaken on appropriate variables; t Tests were conducted to compare PaCKS scores across gender. An analysis of variance (ANOVA) was conducted to analyse how age, country of birth, marital status, household income, and qualifications performed on the PaCKS.

Phase two data were stored and managed through NVivo Software (V13). Qualitative data were transcribed, and any identifying information was removed. Transcripts were analysed using thematic analysis [39] which involved a six-step process: familiarisation, generating codes, searching for themes, reviewing themes, defining and naming themes, and producing a report. Themes were derived by exploring patterns, similarities and differences within and across the data in relation to participant’s awareness, knowledge, and perceptions of palliative care. Data analysis was done by two authors (OB and LH-T) and discussed with a third author (FH) to enhance credibility and trustworthiness.

### Integration

Integration was evident through data transformation between phase one and two [40], and merging in the results and discussion [33]. The data from phase one informed the development of the interview schedule utilised in phase two, and the results from both phases were analysed in parallel, and integrated using data matrix and weaving the thread techniques, and presented thematically throughout the results and discussion.

## Results

### Description of the sample

A total of 2161 people were contacted, 1201 of whom completed the Northern Ireland Life and Times survey (response rate 56%), representative of the demographic profile in NI. The participants were aged between 18 and 95 years (mean: 61 yrs). The largest proportion of the population, 17.7% (*n* = 210) were aged between 45 and 54 years. Over half were female (58.3% *n* = 700); most were white (95.5% *n* = 1147) and born in Northern Ireland (84.2% *n* = 1011). Over two fifths of those surveyed were married (43.3% *n* = 514), described the area where they lived as a ‘small city or town’ (41.5% *n* = 498) and in terms of religion identified themselves as protestant (43.9% *n* = 496). Regarding participant’s level of educational attainment, over a quarter (25.3% *n* = 302) indicated that they had a degree or higher qualification, and just under a quarter (24.7% *n* = 295) reported no formal qualifications. The demographic profile of the survey respondents reflects that of the general population of Northern Ireland [41].

Participants were asked if they had a physical or mental health condition that had lasted or was expected to last for 12 months or more; a quarter of participants (25.6% *n* = 307) acknowledged that they had a physical or mental condition or illness. Of those participants, 58% (*n* = 178) indicated that the condition/illness reduced their ability to carry out day-to-day activities a lot. Only 8.1% (*n* = 97) of participants indicated that they had someone living with them, for whom they had a caring responsibility. A further 9.6% (*n* = 115) of participants had a caring responsibility for a sick, disabled or elderly relative or friend not living with them.

Twenty-five participants took part in phase two. Almost all participants (96%) were white; 60% were male and nearly three quarters (72%) were married or co-habiting. Less than a quarter (24%) of those who participated were under 50 under 50 years old, with the largest proportion of participants (36%) aged between 61 and 70 years. Almost half (48%) were retired, and all participants were Christian.

### Factor analysis of Palliative Care Knowledge Scale (PaCKS)

Thirteen items relating to knowledge of palliative care were subject to factor analysis, extraction method: Maximum Likelihood, with Varimax rotation. The analysis yielded one factor explaining a total of 62.93% of the variance for the entire set of variables. The communalities of the variables included were good and each item included in the one factor model was well-represented with adequate factor loadings (.60–.87). A Chi-square (goodness-of-fit) test was significant indicating that the model results fit the data X_2_ (65, *N* = 1201) 931.18, *p* = < 0.05. The results confirm a single factor structure and justification for the totalling of all 13 scores. Higher scores indicate higher levels of knowledge. Factor loadings and communalities are reported for each item (see Supplementary File 3).

### Demographic variations across palliative care knowledge (PaCKS)

A linear model found significant impact of the following variables on knowledge of palliative care scores; qualification (predictor importance 0.45); description of area lived (predictor importance 0.12); gender (predictor importance 0.09); marital status (predictor importance 0.09); age (predictor importance 0.08); country of birth (predictor importance 0.04). (see Table 2 for mean scores).

- Education had a significant impact on scoring across the PaCK’s total score. Respondents with higher levels of education had better knowledge of palliative care. An ANOVA revealed a significant difference depending on level of education [F (5,1187) = 24.474, *p* = < 0.005); with post hoc tests revealing that those with a degree level qualification or higher, scored significantly higher than those with school qualification (grades A-C) (*p* = < 0.005); and those with school qualification (grades D-G) (*p* = < 0.005).
- An independent samples t-test was conducted to compare PaCKS knowledge scores across gender. There was a significant difference in PaCKS scores between males, mean = 7.67, SD =5.06, and females, mean = 8.77, SD =4.75, t (1199) = − 3.845, *p* < .001. Our results indicated that females had significantly higher palliative care knowledge scores than males. Marital status also had a significant impact on PaCKS scores.
- An ANOVA revealed a significant difference across marital status [F (4,1183) = 15.3, *p* = < 0.005), with post hoc tests revealing that there was a significant difference in knowledge scores between those who were single and those who were married and living with husband/wife (*p* = < 0.001), with married people having a better knowledge score than those who were single; there was also a significant difference between those who were married and living with husband/wife, they scored higher, when compared to those who were married and separated from husband/wife (*p* = < 0.001) and those who were widowed (*p* = < 0.001). Knowledge of palliative care also increased with age.
- An ANOVA revealed a significant difference across age [F (7,1180) = 9.193, *p* = < 0.005], with post hoc tests revealing that there were significant differences in knowledge scores between younger and older populations; with older, for e.g. those aged 18-24 yrs. scored significantly lower than those aged 45-54 yrs. (*p* = < 0.005).

**Table 2:** Participant’s responses to PaCKS items on Knowledge of Palliative Care

Palliative Care Items (PaCKS)	Correct Response	Accurate Statement, n (%)	Inaccurate Statement, n (%)
A goal of palliative care is to address any psychological issues brought up by serious illness	True	694 (57.8%)	507 (42.2%)
Stress from serious illness can be addressed by palliative care	True	760 (63.3%)	441 (36.7%)
Palliative care can help people manage the side effects of their medical treatments	True	863 (71.9%)	338 (28.1%)
When people receive palliative care, they must give up their other doctors	False	759 (63.2%)	442 (36.8%)
Palliative care is exclusively for people who are in the last 6 months of life	False	536 (44.6%)	665 (55.4%)
Palliative care is specifically for people with cancer	False	776 (64.6%)	425 (35.4%)
People must be in the hospital to receive palliative care	False	812 (67.6%)	389 (32.4%)
Palliative care is designed specifically for older adults	False	812 (67.6%)	389 (32.4%)
Palliative care is a team-based approach to care	True	850 (70.8%)	351 (29.2%)
A goal of palliative care is to help people better understand their treatment options	True	796 (66.3%)	405 (33.7%)
Palliative care encourages people to stop treatments aimed at curing their illness	False	730 (60.8%)	471 (39.2%)
A goal of palliative care is to improve a person’s ability to participate in daily activities	True	726 (60.4%)	475 (39.6%)
Palliative care helps the whole family cope with a serious illness	True	870 (72.4%)	331 (27.6%)

### Findings from thematic synthesis

Five overarching themes were identified through the merging and integration of qualitative and quantitative data; three which examined public awareness, knowledge, and perceptions of palliative care and two identified strategies to raise awareness: “Prior knowledge of palliative care”; “Variable understanding of palliative care”; “Promoting public awareness of palliative care”; “Shortcomings in current palliative care information and provision”; and "Future strategies to improve understanding of palliative care".

### Prior knowledge of palliative care

Of those who completed the survey, almost half of participants (44.6%) had some direct experience of palliative care through a friend/relative. A small proportion of the population sampled (11.2%) had a job that involved working with people who received palliative care and three participants (0.3%) were currently receiving palliative care. A fifth of participants (20.1%) indicated that they had previously heard about palliative care from either TV, newspapers or social media, with a further 9% acknowledging that they had heard the term from another source including school/university, word of mouth, a friend/relative, or a medical practitioner. For 13.8% of participants, the term palliative care was familiar, but they were either unsure or could not remember where they had heard the term. Only 14.2% of participants indicated that they had no prior knowledge of the term palliative care.

Similar sources of information on palliative care were reflected in the qualitative data. Participants discussed the diverse sources from which they had gained an understanding of palliative care, including online, media, friends and family, GP and other healthcare professionals and religious and social service providers. However, it was clear from the qualitative findings that respondents’ knowledge and understanding were largely attributed to personal experience. Findings showed that in some instances, these personal experiences led to a negative view of palliative care as this was mostly associated with the end of life care phase. Several respondents focused on medication in the final stages of death and resuscitation options, highlighting ‘*a good death’* and *‘dying comfortably’* as the optimum goals.

### Variable understanding of palliative care

The mean PaCKS score was 8.31 (standard deviation [SD] = 4.91, range 0–13). Correct responses ranged from 44.6–72.4%. Overall, just over a fifth of participants (22.6%) were completely accurate in their understanding of palliative care, scoring a total of 13 out of 13 items correctly. Similarly, just under a fifth (19.5%) answered none of the items correctly. The median score for the sample was 10; indicating that half the sample held at least three misconceptions about palliative care. The most commonly held misconceptions about palliative care included: (PaCKS5) Palliative care is exclusively for people who are in the last 6 months of life (55.4% answered incorrectly); (PaCKS1) A goal of palliative care is to address any psychological issues brought up by serious illness (42.2% answered incorrectly); (PaCKS12) A goal of palliative care is to improve a person’s ability to participate in daily activities (39.6% answered incorrectly); (PaCKS11) Palliative care encourages people to stop treatments aimed at curing their illness (39.2% answered incorrectly); (PaCKS4) When people receive palliative care, they must give up their other doctors (36.8% answered incorrectly); (PaCKS2) Stress from serious illness can be addressed by palliative care (36.7% answered incorrectly); and finally, (PaCKS6) Palliative care is specifically for people with cancer (35.4% answered incorrectly) (See Table 2).

Findings from the qualitative data showed that most of the respondents had some prior knowledge of palliative care while a few participants admitted that they had never heard of the term. This re-enforced findings from the quantitative data. Many of the respondents acknowledged that palliative care was complex and specialised. However, there were varying levels of understanding among respondents when asked to clarify when exactly palliative care took place and who was involved. A number of respondents noted that palliative care happened *‘at the very end’* when there was *‘no hope’*, or no other *‘treatments*’ available. Most respondents agreed that palliative care involved different healthcare professionals (GP, hospital doctors and nurses, hospice nurses, social workers etc) but most struggled with clarifying the various individualised care packages and the range of support available. This highlighted that although there was relatively high awareness of the term palliative care, most respondents had very limited understanding of the term.

### Promoting public awareness of palliative care

In phase two, respondents were asked to give their views of the barriers and facilitators to promoting public awareness of palliative care. Many respondents acknowledged the *‘taboo’* that still existed around public discussions about death and dying, and that in some instances it can be very difficult to broach the subject, even with family and friends. Some respondents acknowledged that relationships and dynamics can be complicated, and that people do not want to cause *‘upset or distress’* by speaking about *‘their own mortality’*. One respondent stated;

*‘It’s never really talked about to be quite honest … like deaths and funerals – nobody really likes to envisage the end … it’s inevitable at some stage, but it’s sort of you don’t talk about it, it’s not going to happen, so to speak’.* (PCACPI003)

Participants advised that opening the conversation of palliative care to a wider audience also means considering the cultural differences that exist amongst the public in terms of their views, understanding, and the role of palliative care within end of life care. Many respondents who were interviewed openly acknowledged that their religious or cultural beliefs dictated the decisions they make in terms of their care, both physical and spiritual. A number of respondents discussed resuscitation, organ donation, cremation etc. all through the lens of their personal beliefs and were largely unconcerned with the idea of doing something different/contrary to what they were familiar with. A number of respondents also highlighted the limitations of human knowledge and technological advancement, and that our actual time of death is beyond the scope of human prediction; ‘ … *the idea that there is no point almost in worrying about what was going to happen. If it’s going to happen anyway* … ’ (PCACPI002).

### Shortcomings in current palliative care information and provision

Many respondents who took part in phase two highlighted that information being provided on palliative care to the public had to overcome the challenging attitudes that existed. Informing people about palliative care involves facilitating individuals to realise and acknowledge their own mortality as well as the importance of taking responsibility for their own health and wellbeing, whilst they still have the capacity to do so. One respondent noted that palliative care could be included in something like life insurance, that way, people availed of it (and learned about the benefits) in the context of living and paying for their mortgage but knowing that they’d be taken care of in the future.

*‘It’s bringing it into the discussion, it’s creating a solution. How you do it? Like I say … a certain percentage of people have life insurance. If you’ve got a mortgage, you need life insurance … but palliative care isn’t something that has been spoken about. If I’m diagnosed with a terminal illness, the house is paid off if I’m going to die. If you are not in a position where you even think you’re going to avail of palliative care at any stage in the near future, you’re not going to think about it’.* (PCACPFG002).

### Future strategies to improve understanding of palliative care

Respondents suggested various approaches for the dissemination of information on palliative care via a variety of platforms. More traditional methods discussed included information in GP surgeries, libraries, posting leaflets, traditional media platforms (e.g. T.V., radio, newspapers and billboards) alongside the use of media and television such as soap storylines. Respondents also spoke about the internet and social media and their role as a global information resource. Many agreed that such online platforms were better able to reach younger generations, but some cautioned being able to trust ‘*everything you see online’*, indicating that some regulation would be required to ensure that the information people were searching for was accurate.

There was an overarching feeling that this information and the need to educate people was ultimately about ‘*supporting them’* to make informed choices and decisions about their lives and their death, if that was their wish. This was important to both move beyond awareness and seek to enhance understanding.

## Discussion

Whilst the findings indicate the public may claim to be aware of the term palliative care, there is an inadequate understanding of the concept, with only a fifth of the sample obtaining full scores. The current study identified 14.2% of participants had no knowledge of palliative care. This is consistent with previous international [12, 20–22, 24, 27], and national [10, 15, 16, 28] literature. For example, a study undertaken in Northern Ireland that reported 19% of the 600 members of the public who completed a cross-sectional survey had no understanding of palliative care and a further 56% had very low knowledge [15]. This also correlates with an American study which used the PaCKS tool and found limited understanding or palliative care (mean score of 5.25), with a significant proportion of the 301 participants responding “I don’t know” for every item [20]. The timing of these studies would also suggest that understanding is not improving, despite policy initiatives, media and wider public engagement strategies.

Personal experience **s**haped many participants’ views on palliative care and potentially their misconceptions, which is also a common thread in the literature [11, 15, 27]. For example, over half of the participants in Shalev et al’s [21] study held a misperception about palliative care and were not aware of the major components. The most common misconception about palliative care in this study was that it is exclusively for people in the last 6 months of life. This is supported by previous literature internationally, which repeatedly reports a public perception of palliative care provided at the very end of life [11, 15, 26]. Internationally, it is also reflected in health systems insurance policy, were palliative care is provided 6 months prior [42] Other common misconceptions included a tendency to associate palliative care for those diagnosed with cancer, a focus on pain relief whilst other aspects such as spiritual care were not mentioned.

In this study, being female, higher educated, married, and older, were significant factors that influenced level of awareness and the number of correct responses. Similar findings have been noted internationally, including from research in America [21]; Saudi Arabia [24]; Nigeria [27]; Italy [12]; Sweden [22]; and the UK [10, 15, 16, 28]. These studies have consistently demonstrated that palliative care awareness among the general public is variable and that demographic factors have a significant impact. Whilst this may provide a focus for future initiatives to be based upon, it is however unclear why such disparities exist and the impact of these on palliative care receptivity.

Recent models of palliative care advocate earlier integration of palliative care in public health yet evidence would indicate that this is not happening in practice [6]. According to Collins [9] one of the potential contributing factors to this is the general public’s attitude and level of knowledge about palliative care. Despite respondents in the current study advocating the need to hold open conversations about palliative care in society, they had concerns about the social taboo of talking about death and fear of causing upset. Qualitative comments highlighted that the public’s exposure to palliative care was centred around the end of life period with the introduction of specialist health care professionals and services. Without open conversations and readily available information, such experiences provided a framework upon which to articulate and base their understandings of what constitutes palliative care. This highlights issues that Collins et al. [9] consider as an ‘image/language problem’ for palliative care. Such misconceptions should not be dismissed as they may deter people from accessing services in the future. For example, if the public view palliative care as predominately for people diagnosed with non-curative cancer this may lead to a false impression that this service is not appropriate for themselves or their loved ones. The need therefore for public health campaigns to recognise this disparity in what palliative care is seen to offer, from the public’s perspective, needs to be recognised and responded to. This would be in line with the EIU Quality of Death Index’s [43] that recommends that perceptions of death and cultural taboos are confronted to improve end of life outcomes.

To move forward from these findings and raise awareness, remove misconceptions, and increase openness to holistic palliative care, a generic recommendation from previous studies and reaffirmed in this study is the need for a public education programme. There are two key strands required within this public education programme; the normalisation of palliative care and addressing misperceptions and knowledge gaps. Normalisation requires a structured and systemic introduction of palliative care into the context of everyday life, such as through life insurance and mortgage applications. It was not seen as the sole remit of healthcare professionals; instead education, media, and individuals all were seen to hold personal responsibility to contribute to an open dialogue. To address misperceptions and knowledge gaps, a consistent message from a trustworthy source, inclusive of the voice of the patient, carer, and healthcare professional, and offering both general and tailored information to the needs of specific groups (gender, age, rural/urban communities) is advocated. It is also vital to consider some essential messages of the educational intervention. Building on the work of Collins [9], an increasing evidence base, and drawing from aspects of the PaCKS tool some of the following essential components of educational message are proposed (see Table 3). Yet to achieve this there is a need for service capacity to also be reviewed to enable palliative care to be integrated earlier.

**Table 3:** Essential Messages of Educational Message for Palliative Care

Essential Messages	
Palliative care considered as a system of ‘best care’, not linked to specific ‘place’ or setting	
Viewed as an active approach to care, offering solutions and improving quality of life	
Enabling people to stay out of hospital and provided across all settings, all conditions and all times.	
Enabling choices, decision making and facilitating goals of care for both patients and families	
Providing expert management of symptoms from members of specialist and generalist multidisciplinary team	
Facilitation of living independently as well as possible for as long as possible	
Earlier integration in the patients’ journey and includes but not just focused on end of life care.	

### Study limitations

Whilst this study adopted a mixed methods approach with phase one based on a random sample representative of the total Northern Ireland population [36], the structured framework of the NILT survey did not allow to fully explore why people think the way they do and only provides a snap shot of attitudes for that period in time. Using a qualitative approach helped to contribute to a broader understanding of the public’s views, however, 48% of the sample were retired, and everyone identified as white and Christian, hence bias may be introduced. A number of researchers from different background and specialties were involved in data collection which may introduce a source of researcher bias. To manage this bias, data analysis was completed by multiple researchers and reviewed by a team member separate from the data collection process.

## Conclusions

In conclusion, this population based mixed methods study builds upon previous research in this area, indicating widespread misunderstandings of the concept palliative care. Variances in understanding were associated with several demographic characteristics however reasons why these are influencers are unknown. Whilst the public report a willingness to engage in such conversations, societal restrictions impinge on this occurring. Understanding is derived from limited ad hoc personal experiences focusing on the end of life and not the holistic palliative care journey. The findings provide an empirical basis from which to understand how the public view palliative care, to inform and stimulate focused debate on how to increase awareness and dispel misunderstandings. Education is key to advancing public understanding of palliative care. By shifting the view of palliative care to earlier integration across disease types and care settings, essential conversations can start earlier and ensure palliative care reaches everyone who would benefit.

## Supplementary Information

**Additional file 1: Supplementary information 1.** Questionnaire. **Supplementary information 2.** Interview Schedule. **Supplementary information 3.** Factor Analysis Table for PaCKS.

## Data Availability

The datasets generated during and/or analyzed during the current study are not publicly available but are available from the corresponding author on reasonable request.

## Abbreviations

ANOVA: An analysis of variance
HCP: Healthcare Professional
NILT: Northern Ireland Life and Times
PaCKS: Palliative Care Knowledge Scale
SREC: School Research Ethics Committee
WHO: World Health Organization
