# Supplementary material for: Examining public knowledge, attitudes and perceptions towards palliative care: a mixed method sequential study
Source: BMC Palliat Care. 2021 Mar 17;20:44. doi: 10.1186/s12904-021-00730-5 (PMC7971949; doi:10.1186/s12904-021-00730-5)
Supplement: Supplementary file 1 — Additional file 1: Supplementary information 1. Questionnaire. Supplementary information 2. Interview Schedule. Supplementary information 3. Factor Analysis Table for PaCKS. [file 12904_2021_730_MOESM1_ESM.pdf]

SUPPLEMENTARY INFORMATION 1

Questionnaire

SECTION G: PALLIATIVE CARE

The next set of questions are about palliative care. This can be a sensitive topic for many people. We need to try and hear as many views as possible, however if at any time, talking about these things is upsetting, please let me know and we can skip to the end of these questions.

G1 I am going to read out some statements about palliative care. For each statement, please tell me if you think the statement is true or false
READ OUT
SINGLE CODE PER STATEMENT

	True	False	Don't know
PACKS1<br>A goal of palliative care is to address any psychological issues brought up by serious illness	1	2	8
PACKS2<br>Stress from serious illness can be addressed by palliative care	1	2	8
PACKS3<br>Palliative care can help people manage the side effects of their medical treatments	1	2	8
PACKS4<br>When people receive palliative care, they must give up their other doctors	1	2	8
PACKS5<br>Palliative care is exclusively for people who are in the last 6 months of life	1	2	8
PACKS6<br>Palliative care is specifically for people with cancer	1	2	8
PACKS7<br>People must be in the hospital to receive palliative care	1	2	8
PACKS8<br>Palliative care is designed specifically for older adults	1	2	8
PACKS9<br>Palliative care is a team-based approach to care	1	2	8
PACKS10<br>A goal of palliative care is to help people better understand their treatment options	1	2	8
PACKS11<br>Palliative care encourages people to stop treatments aimed at curing their illness	1	2	8
PACKS12<br>A goal of palliative care is to improve a person's ability to participate in daily activities	1	2	8
PACKS13<br>Palliative care helps the whole family cope with a serious illness	1	2	8

G2 From where, if at all, had you previously heard of the term 'palliative care'?
CODE ALL THAT APPLY

HEARDPC1	I had never heard of the term	1
HEARDPC2	A friend/ relative received Palliative Care	2
HEARDPC3	My job involves/involved working with people who receive Palliative Care	3
HEARDPC4	I am receiving Palliative Care	4
HEARDPC5	I heard about it from TV, newspapers and social media	5
HEARDPC6	I heard about it from another source (Please write in)	6
HEARDPCDK	Not sure/ can't remember	8

SUPPLEMENTARY INFORMATION 2

Interview Schedule
Study title: INFORMING THE DEVELOPMENT OF AN EVIDENCE BASED PUBLIC HEALTH APPROACH: PUBLIC AWARENESS OF PALLIATIVE CARE - PHASE 2

Topic themes & questions

General knowledge of palliative care

- Could you please describe for me what you think palliative care is?
- Where do you think palliative care takes place?
- Views and opinions of palliative care.

Knowledge and information

- If you needed information about palliative care, where would you look for it, or whom would you ask?

Accessibility

- Where do you think people go to get palliative care services?

Future Strategies

- What do you think are the supporting factors for promoting public awareness of palliative care?
- What do you think are the inhibiting factors for promoting public awareness of palliative care?
- What could be done to promote more openness in discussion (and to inform future strategies)?
- Would you like to see palliative care publicly promoted? Please explain. If yes how would this be achieved.

Warm down/ Debrief exercise

- Reflect on main issues discussed and remind the participant of confidentiality and information and support services available, final thank you. Hand out a support pack to every participant.

SUPPLEMENTARY INFORMATION 3

Factor Analysis Table for PaCKS

**PaCKS Item**	**Factor Loadings**	**Communalities**
**1**.A goal of palliative care is to address any psychological issues brought up by serious illness	.686	.471
**2.**Stress from serious illness can be addressed by palliative care	.764	.583
**3.**Palliative care can help people manage the side effects of their medical treatments	.770	.592
**9**.Palliative care is a team-based approach to care	.870	.757
**10.**A goal of palliative care is to help people better understand their treatment options	.729	.531
**12**.A goal of palliative care is to improve a person's ability to participate in daily activities	.745	.555
**13.**Palliative care helps the whole family cope with a serious illness	.884	.782
**4.**When people receive palliative care, they must give up their other doctors	.774	.598
**5**.Palliative care is exclusively for people who are in the last 6 months of life	.597	.356
**6.**Palliative care is specifically for people with cancer	.808	.653
**7.**People must be in the hospital to receive palliative care	.868	.753
**8.**Palliative care is designed specifically for older adults	.846	.716

**11**.Palliative care encourages people to stop treatments aimed at curing their illness	.763	.582
